# Development and validation of an echocardiographic nomogram for identifying cardiac amyloidosis in patients with left ventricular hypertrophy

**DOI:** 10.1186/s12872-025-04973-5

**Published:** 2025-10-27

**Authors:** Shichu Liang, Zhiyue Liu, Fanfan Shi, Liping Chen, Dayan Li, Ying Peng, Wenfeng He, Chaohui Du, He Huang

**Affiliations:** 1 Department of Cardiology, West China Hospital, Sichuan University, No.37 GuoXue Alley, Chengdu, 610041 China; 2 Department of Clinical Research and Management, Center of Biostatistics, Design, Measurement and Evaluation (CBDME), West China Hospital, Sichuan University, Chengdu, China; 3 Department of Cardiology, Shang Jin Hospital of West China Hospital, Sichuan University, Chengdu, China; 4 Department of Cardiology, West China Tianfu Hospital, Sichuan University, Chengdu, China

**Keywords:** Cardiac amyloidosis, Left ventricular hypertrophy, Echocardiography, Nomogram

## Abstract

**Background:**

Echocardiography is the principal non-invasive imaging modality for screening cardiac amyloidosis (CA). This study aimed to establish a cohort of CA-associated left ventricular hypertrophy (CA-LVH) within a hospital-based population and to develop an echocardiographic identification model for CA using readily available echocardiographic parameters.

**Methods:**

This retrospective nested cohort study involved the collection of clinical and echocardiographic data from three hospitals affiliated with the West China Medical Center, Sichuan University, between January 1, 2008, and December 31, 2023. The relative wall thickness (RWT) was calculated as twice the left ventricular posterior wall thickness (LVPW) divided by the left ventricular internal diameter (LVID). Asymmetric hypertrophy was defined as a ratio of interventricular septal thickness (IVS) to LVPW greater than 1.3. The AMYLI score was computed as the product of RWT and E/e’ ratio.

**Results:**

A total of 185 CA patients (183 AL-CA and 2 ATTR-CA) who underwent 309 echocardiography examinations from different time periods with 1,213 echocardiographic data points from in-hospital non-CA-LVH cases matched for age, gender, and body surface area were included. Multivariable logistic regression analysis identified a history of hypertension [odds ratio (OR): 0.04, 95% confidence interval (CI): 0.021–0.073], LVID [OR: 0.927, 95%CI: 0.878–0.977], left ventricular ejection fraction (LVEF) [OR: 0.95, 95%CI: 0.908–0.993], AMYLI score [OR: 1.088, 95%CI: 1.024–1.161], asymmetric hypertrophy [OR: 3.729, 95%CI: 1.884–7.441], granular sparkling [OR: 3.111, 95%CI: 1.355–7.431], small pericardial effusion [OR: 2.77, 95%CI: 1.563–4.937], mild aortic regurgitation [OR: 2.353, 95%CI: 1.278–4.361], mild mitral regurgitation [OR: 4.331, 95%CI: 2.347–8.141], and mild tricuspid regurgitation [OR: 3.837, 95%CI: 2.026–7.358] as independent predictive factors for CA in LVH patients. The predictive factors were used to construct a nomogram model, which demonstrated high accuracy (0.91–0.92), specificity (0.91–0.92), sensitivity (0.90–0.91), positive predictive value (0.73), negative predictive value (0.93–0.98), and Youden index (0.81–0.83).

**Conclusion:**

The developed nomogram displayed remarkable predictive accuracy, which has the potential to enhance CA screening via routine echocardiography and strategically guide subsequent diagnostic evaluations.

**Supplementary Information:**

The online version contains supplementary material available at 10.1186/s12872-025-04973-5.

## Introduction

Cardiac amyloidosis (CA) is an infiltrative cardiomyopathy characterized by the progressive deposition of insoluble amyloid protein fibrils within the myocardial extracellular space, eventually leading to the disruption of normal tissue architecture and function [1]. Its predominant CA includes immunoglobulin light-chain CA (AL-CA) and transthyretin CA (ATTR-CA) [2]. Amyloid protein fibrils affect various organs and systems, including the spine, kidneys, gastrointestinal tract, and nervous system, leading to a diverse range of clinical presentations and complicating diagnoses [3]. Notably, CA is associated with a high risk of progression to refractory heart failure, with a poor prognosis [4]. However, early detection of CA remains challenging, highlighting the need for early diagnosis and treatment to optimize patient outcomes [3].

Echocardiography is the preferred non-invasive imaging modality for cardiac assessment and serves as the principal screening tool for CA, facilitating the diagnosis of CA and guiding subsequent examinations [5]. In the 2022 European Society of Cardiology (ESC) Cardio-Oncology guidelines, echocardiography is recommended as a Class IB tool for the diagnosis of CA [4]. The “red flags” of CA, such as ventricular wall thickening, biatrial enlargement, and diastolic dysfunction, are valuable for diagnosis [6]. In recent years, two-dimensional speckle tracking strain analysis has garnered extensive attention for CA screening [7]. While strain measurement is readily available for CA screening, it is underutilized in most centers, particularly in primary-level hospitals [8]. In primary-level hospitals that serve large populations, more patients may benefit from routine echocardiographic examinations for CA screening.

Of note, left ventricular hypertrophy (LVH) is the most prevalent finding in CA and is a key indicator for clinical suspicion and diagnosis of CA. It has been reported that about 5% of patients initially diagnosed with hypertrophic cardiomyopathy (HCM) are actually suffering from ATTRm-CA [1]. Previous researches have primarily focused on the clinical presentations and functional changes in CA. This study aimed to establish a CA-LVH cohort from hospital-based populations, focusing on routine echocardiographic parameters readily accessible in CA patients, to provide methodological support for early screening and to develop a simple, convenient diagnostic tool for CA in the Asian population.

## Methods

### Study design and population

This investigation utilized a nested retrospective cohort design to assess the echocardiographic characteristics of patients diagnosed with LVH across three hospitals of the West China Medical Center, Sichuan University (West China Hospital, Shang Jin Hospital of West China Hospital, and West China Tianfu Hospital, Sichuan University).

The study enrolled inpatients who had undergone echocardiography and were diagnosed with LVH between January 1, 2008, and December 31, 2023. Data were collected from the electronic medical records of West China Medical Center, Sichuan University. Following data extraction, a cohort focusing on CA-LVH echocardiography was established based on predefined inclusion and exclusion criteria. This study was approved by the Biomedical Research Ethics Committee of West China Medical Center, Sichuan University (Ethics approval number: 20221448).

#### Inclusion criteria

(1) Patients aged 18 years or older; (2) Individuals who had undergone echocardiography at West China Medical Center, Sichuan University and were diagnosed with LVH; (3) Echocardiography demonstrating a left ventricular ejection fraction (LVEF) of 50% or greater.

#### Exclusion criteria

(1) Patients who had undergone cardiac surgery or percutaneous cardiac intervention, had moderate-to-severe valvular stenosis, congenital heart disease, a history of coronary atherosclerotic heart disease with abnormal wall motion, acute heart failure, or hypertrophic obstructive cardiomyopathy; (2) Patients with poor echocardiographic image quality or incomplete data that precluded analysis.

The case group was defined as LVH patients diagnosed with CA in accordance with the 2021 ESC and 2022 American Heart Association (AHA)/American College of Cardiology (ACC)/Heart Failure Society of America (HFSA) guidelines [9, 10]. In cases where pathological validation of amyloidosis was not feasible due to negative biopsy outcomes or the inability of patients to undergo biopsy, a comprehensive evaluation of clinical manifestations, electrocardiographic tracings, echocardiographic assessments, cardiac magnetic resonance (CMR) imaging, and laboratory findings, supported by a multidisciplinary expert consensus, facilitated the clinical diagnosis of CA [11]. The control group included in-hospital LVH patients, who were eventually diagnosed with hypertensive heart disease (HHD) [12], HCM [13, 14], uremic cardiomyopathy (UM) [15], Fabry disease (FD) [16], and LVH of unknown origin. All patients from control group were ruled out the possibility of AL-CA or ATTR-CA according to the clinical, biological and imaging examinations during their hospitalization.

### Definition of left ventricular hypertrophy

LVH was diagnosed according to the 2015 guidelines issued by the European Association of Cardiovascular Imaging in collaboration with the American Society of Echocardiography (ASE) [17]. Echocardiographic assessments focused on key parameters, including left ventricular internal diameter (LVID), interventricular septal thickness (IVS), and left ventricular posterior wall thickness (LVPW) at end-diastole. The diagnosis of LVH was confirmed when the left ventricular mass index (LVMI) exceeded 95 g/m^2^ for females and 115 g/m^2^ for males. LVMI was calculated by dividing the left ventricular mass (LVM) by the body surface area (BSA). The formulas for calculating LVM and BSA are as follows [18]:

$$\mathrm{LVM}\;(\mathrm g)\;=\;0.8\;\mathrm \times\;1.04\;\lbrack{(\mathrm{LVID}\;+\;\mathrm{LVPW}\;+\;\mathrm{IVS})}^3\;-\;(\mathrm{LVID})^3]+\;0.6;$$

$$\mathrm{BSA}\;(\mathrm m^2)\;=\;0.0061\;\mathrm \times\;\mathrm{height}\;(\mathrm{cm})\;+\;0.0128\;\mathrm \times\;\mathrm{weight}\;(\mathrm{kg})\;-\;0.1529;$$

$$\mathrm{LVMI}\;(\mathrm g/\mathrm m^2)\;=\;\mathrm{LVM}\;(\mathrm g)\;/\;\mathrm{BSA}\;(\mathrm m^2).$$

### Clinical evaluation and echocardiographic tests

Clinical data collected for each patient comprised age, gender, height, weight, and history of hypertension. In the present study, the echocardiographic data were recorded by cardiologists with advanced training in echocardiography (with over five years of echocardiography experience). Meanwhile, the echocardiograms were carried out by experienced sonographers in accordance with the guidelines of the ASE [17]. Cardiologists who performed the echocardiography were blinded to the results of other assessments.

Echocardiographic measurements were performed in the parasternal long-axis view to assess the left atrial diameter (LAD), LVID, right ventricular diameter (RVD), IVS, and LVPW. The apical four-chamber view was utilized to measure the right atrial diameter (RAD) [17]. The Simpson’s biplane method was employed to measure end-diastolic volume (EDV), end-systolic volume (ESV), stroke volume (SV), and LVEF [17]. The relative wall thickness (RWT) was calculated as twice the LVPW divided by the LVID [17]. Asymmetric hypertrophy was defined as a ratio of IVS to LVPW greater than 1.3 [19].

Mitral valve inflow was evaluated in the apical four-chamber view, recording the early diastolic mitral valve peak inflow velocity, E-wave (E) [17]. Tissue Doppler imaging was utilized to assess mitral annulus movement, determining the early diastolic maximum velocities of the septal e’ and lateral wall e’, and calculating the E/e’ ratio [E/e’ = 2 x E/(septal e’ + lateral e’)] [17]. Measurements were performed in triplicate and then averaged. The AMYLI score was computed as the product of RWT and E/e’ ratio [20]. Aortic forward flow velocity (aortic velocity, AV) was recorded from the apical five-chamber view [17].

The presence of granular sparkling was re-evaluated by experienced echocardiographers from West China Hospital, Sichuan University (Zhiyue Liu and He Huang) who were blinded to the result of the reported findings. The degree of pericardial effusion was categorized as small, moderate, or large, following the 2015 ESC criteria for pericardial diseases [21]. Mitral regurgitation (MR), tricuspid regurgitation (TR), and aortic regurgitation (AR) were classified as mild, moderate, or severe, in accordance with the ASE guidelines [22].

### Statistical analysis

Statistical analysis was conducted using R (version 4.2.2). Data normality was assessed using the Kolmogorov-Smirnov test. Quantitative variables with a normal distribution were reported as mean ± standard deviation and compared using the t-test. Variables with non-normal distributions were presented using the median and interquartile range and compared using the Mann-Whitney U test. Qualitative variables were expressed as percentages and compared using the chi-square test or Fisher’s exact test. Propensity score matching (PSM), using the nearest neighbor technique, was applied to create a 1:4 match between CA and non-CA patients, with a caliper of 0.05 [23]. Internal validation was performed in accordance with TRIPOD guidelines, with subjects randomly allocated into a 7:3 ratio for training and validation sets [24].

Initially, univariate logistic regression was performed to identify factors predicting CA, which were subsequently incorporated into the multivariate model. Effect sizes were quantified as odds ratios (OR) and 95% confidence intervals (CI). Next, a nomogram was developed based on this model. Calibration curve analysis was performed using internal validation with 1,000 bootstrap resamples. Model performance was evaluated using the area under the receiver operating characteristic (ROC) curve (AUC) and concordance index (C index), where a C index ≥ 0.70 indicated strong discrimination. Calibration curves were plotted to assess model fit, with ideal performance represented by the 45-degree line. Decision curve analysis (DCA) was conducted to evaluate model utility and clinical applicability. Model performance was compared with existing models using accuracy, sensitivity, specificity, Youden index, positive predictive value, and negative predictive value. A higher Youden index suggests superior diagnostic accuracy [25]. A two-sided *P* < 0.05 was considered statistically significant.

## Results

### Study population

**Fig. 1:**
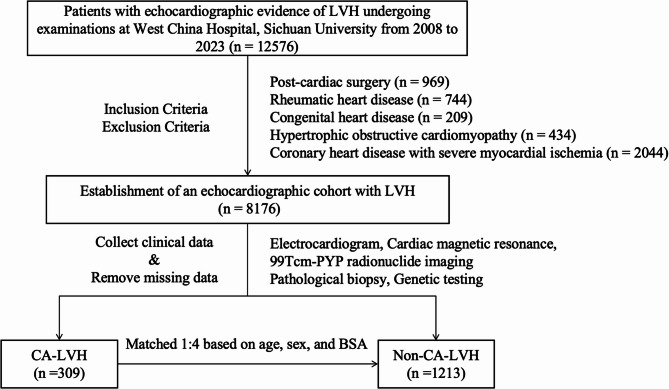
Study flowchart

Between January 1, 2008, and December 31, 2023, a cohort of 12,576 echocardiographic examinations indicating LVH was assembled from the West China Hospital, Shang Jin Hospital of West China Hospital, and West China Tianfu Hospital, Sichuan University. Following the stringent application of inclusion and exclusion criteria, the following cases were excluded: 969 post-cardiac surgery cases, 744 with rheumatic heart disease, 209 with congenital heart disease, 434 with hypertrophic obstructive cardiomyopathy, and 2,044 with severe myocardial ischemia due to coronary heart disease. Consequently, a total of 8,176 routine echocardiographic datasets for LVH patients were included in the analysis. The detailed study flowchart is illustrated in Fig. 1.

Clinical data collection yielded 185 CA patients who underwent 309 echocardiographic examinations from different time periods. The cohort comprised 117 males and 68 females aged between 23 and 87 years. Among them, 183 cases were identified as AL-CA and 2 as ATTR-CA. Histological confirmation of CA was achieved in 103 patients through various biopsy methods, including myocardial (*n* = 27), adipose tissue (*n* = 41), renal (*n* = 25), lip and tongue (*n* = 2), bone marrow (*n* = 2), rectal (*n* = 2), liver (*n* = 1), bladder (*n* = 1), skin (*n* = 1), and lymph node (*n* = 1) biopsies. An additional 82 patients were clinically diagnosed with CA by a multidisciplinary panel of experts based on clinical presentations and supplementary diagnostic tests.

The control group was matched at a 1:4 ratio to the CA group based on age, gender, and BSA, resulting in 1,213 echocardiographic datasets for LVH patients. This group included 488 cases of UM, 463 HHD cases, 80 HCM cases, 2 FD cases, and 180 cases of unexplained LVH. The results of PSM are delineated in Supplemental Fig. 1.

### Demographic and echocardiographic profiles of enrolled patients

Table 1 presents a comprehensive comparison of demographic and echocardiographic features between the CA-LVH and non-CA-LVH groups following a 1:4 matching ratio. Notably, gender, age, and BSA were comparable post-matching. However, the prevalence of hypertension was significantly lower in the CA-LVH group compared to the control group (*P* < 0.05). Echocardiographic analysis revealed significantly lower LVID (43.35 ± 5.54 mm vs 47.39 ± 5.73 mm), lower AV (1.48 ± 0.25 m/s vs 1.31 ± 0.24 m/s), EDV index (54.66 ± 14.89 mL/m^2^ vs 67.04 ± 18.67 mL/m^2^), ESV index (20.35 ± 7.04 mL/m^2^ vs 23.16 ± 9.05 mL/m^2^), SV index (34.30 ± 9.77 mL/m^2^ vs 43.88 ± 11.37 ml/m^2^), LVEF (62.83 ± 6.90% vs 66.00 ± 6.12%), and LVMI (139.20 [120.19, 173.55] g/m^2^ vs 150.92 [131.35, 174.32] g/m^2^) in the CA-LVH group compared to the non-CA-LVH group, and significantly higher LAD (38.85 ± 6.26 mm vs 37.92 ± 5.82 mm), RAD (40.32 ± 7.12 mm vs 36.03 ± 5.72 mm), IVS (15.07 ± 2.97 mm vs 13.83 ± 1.82 mm), LVPW (12.68 ± 2.50 mm vs 12.27 ± 1.26 mm), RWT (0.60 ± 0.16 vs 0.52 ± 0.08), E/e’ ratio (20.00 [14.00, 26.25] vs 15.00 [12.00, 20.00]), and AMYLI score (10.92 [7.40, 17.46] vs 7.59 [5.90, 10.12]), with all *P* < 0.05.

**Table 1:** Comparison of general demographic data and echocardiographic parameters between the two groups

	Before PSM			After PSM		
	CA-LVH (*n* = 309)	Non-CA-LVH (*n* = 7826)	*P* Value	CA-LVH (*n* = 309)	Non-CA-LVH (*n* = 1213)	*P* Value
Demographic characters						
Male (%)	187 (60.5)	5347 (68.3)	0.005	187 (60.5)	758 (62.5)	0.567
Age (years)	61.41 ± 11.78	54.99 ± 16.13	< 0.001	61.41 ± 11.78	61.78 ± 14.69	0.682
BSA (m^2^)	1.58 ± 0.18	1.71 ± 0.21	< 0.001	1.58 ± 0.18	1.59 ± 0.17	0.389
Hypertension History (%)	40 (12.9)	6372 (81.4)	< 0.001	40 (12.9)	989 (81.5)	< 0.001
Echocardiographic parameters						
LVID (mm)	43.35 ± 5.54	48.07 ± 5.57	< 0.001	43.35 ± 5.54	47.39 ± 5.73	< 0.001
LAD (mm)	38.85 ± 6.26	38.22 ± 5.76)	0.059	38.85 ± 6.26	37.92 ± 5.82	0.014
RVD (mm)	21.17 ± 2.90	21.71 ± 2.63	0.001	21.17 ± 2.90	21.41 ± 2.79	0.19
RAD (mm)	40.32 ± 7.12	36.32 ± 5.76	< 0.001	40.32 ± 7.12	36.03 ± 5.72	< 0.001
IVS (mm)	15.07 ± 2.97	14.11 ± 2.31	< 0.001	15.07 ± 2.97	13.83 ± 1.82	< 0.001
LVPW(mm)	12.68 ± 2.50	12.35 ± 1.35	< 0.001	12.68 ± 2.50	12.27 ± 1.26	< 0.001
IVS/LVPW ratio	1.21 ± 0.28	1.16 ± 0.28	0.001	1.21 ± 0.28	1.14 ± 0.21	< 0.001
Asymmetric hypertrophy (%)	84 (27.2)	875 (11.2)	< 0.001	84 (27.2)	124 (10.2)	< 0.001
RWT	0.60 ± 0.16	0.52 ± 0.08	< 0.001	0.60 ± 0.16	0.52 ± 0.08	< 0.001
AV (m/s)	1.31 ± 0.24	1.47 ± 0.25	< 0.001	1.31 ± 0.24	1.48 ± 0.25	< 0.001
E/e’	20.00 [14.00, 26.25]	14.00 [11.67, 18.00]	< 0.001	20.00 [14.00, 26.25]	15.00 [12.00, 20.00]	< 0.001
AMYLI Score	10.92 [7.40, 17.46]	7.28 [5.72, 9.36]	< 0.001	10.92 [7.40, 17.46]	7.59 [5.90, 10.12]	< 0.001
EDV index (ml/m^2^)	54.66 ± 14.89	64.62 ± 18.07	< 0.001	54.66 ± 14.89	67.04 ± 18.67	< 0.001
ESV index(mlm^2^)	20.35 ± 7.04	22.25 ± 8.68	< 0.001	20.35 ± 7.04	23.16 ± 9.05	< 0.001
SV index (mlm^2^)	34.30 ± 9.77	42.38 ± 11.24	< 0.001	34.30 ± 9.77	43.88 ± 11.37	< 0.001
LVEF (%)	62.83 ± 6.90	66.12 ± 6.17	< 0.001	62.83 ± 6.90	66.00 ± 6.12	< 0.001
LVMI (g/m^2^)	139.20 [120.19, 173.55]	145.28 [127.32, 168.56]	0.037	139.20 [120.19, 173.55]	150.92 [131.35, 174.32]	< 0.001
Echocardiographic characters						
Granular sparkling (%)	105 (34.0)	278 (3.6)	< 0.001	105 (34.0)	26 (2.1)	< 0.001
Pericardial effusion (%)	…	…	< 0.001	…	…	< 0.001
None	142 (46.0)	6250 (79.9)	…	142 (46.0)	947 (78.1)	…
Small	156 (50.5)	1443 (18.4)	…	156 (50.5)	254 (20.9)	…
Moderate-to-Large	11 (3.6)	133 (1.7)	…	11 (3.6)	12 (1.0)	…
Aortic Regurgitation (%)	…	…	< 0.001	…	…	< 0.001
None	190 (61.5)	6760 (86.4)	…	190 (61.5)	990 (81.6)	…
Mild	116 (37.5)	1012 (12.9)	…	116 (37.5)	214 (17.6)	…
Moderate-to-Severe	3 (1.0)	54 (0.7)	…	3 (1.0)	9 (0.7)	…
Mitral Regurgitation (%)	…	…	< 0.001	…	…	< 0.001
None	71 (23.0)	6247 (79.8)	…	71 (23.0)	908 (74.9)	…
Mild	227 (73.5)	1444 (18.5)	…	227 (73.5)	286 (23.6)	…
Moderate-to-Severe	11 (3.6)	135 (1.7)	…	11 (3.6)	19 (1.6)	…
Tricuspid Regurgitation (%)	…	…	< 0.001	…	…	< 0.001
None	68 (22.0)	6379 (81.5)	…	68 (22.0)	925 (76.3)	…
Mild	203 (65.7)	1262 (16.1)	…	203 (65.7)	256 (21.1)	…
Moderate-to-Severe	38 (12.3)	185 (2.4)	…	38 (12.3)	32 (2.6)	…

*BSA* Body surface area, *LVID* Left ventricular internal diameter, *LAD* Left atrial diameter, *RVD* Right ventricular diameter, *RAD* Right atrial diameter, *IVS* Interventricular septal thickness, *LVPW* Left ventricular posterior wall thickness, *RWT* Relative wall thickness, *AV* Aortic velocity, *EDV* End-diastolic volume, *ESV* End-systolic volume, *SV* Stroke volume, *LVEF* Left ventricular ejection fraction, *LVMI* Left ventricular mass index, *AR* Aortic regurgitation, *MR* Mitral regurgitation, *TR* Tricuspid regurgitation

Additionally, the incidence of asymmetric hypertrophy (27.2% vs. 10.2%), granular sparkling (34.0% vs. 2.1%), small pericardial effusion (50.5% vs. 20.9%), mild MR (73.5% vs. 23.6%), mild AR (37.5% vs. 17.6%), and mild TR (65.7% vs. 21.1%) was significantly higher in the CA-LVH group, with all *P* < 0.05.

### Comparison between the training and validation sets

Patients were stratified by propensity score and randomly assigned to either the training set or the validation set at a ratio of 7:3. The training set included 1,065 echocardiographic records, encompassing 209 CA cases, 345 μm cases, 326 HHD cases, 58 HCM cases, 1 FD case, and 126 cases of unexplained LVH. On the other hand, the validation set included 457 echocardiographic records, consisting of 100 CA cases, 143 μm cases, 137 HHD cases, 22 HCM cases, 1 FD case, and 54 unexplained LVH cases. The comparison between the training and validation sets is listed in Supplemental Table 1, displaying that demographic or echocardiographic data (all *P* > 0.05) were comparable between the two groups.

### Echocardiographic predictors of cardiac amyloidosis in left ventricular hypertrophy

Univariate logistic regression analysis identified several predictors of CA in patients with LVH, including the absence of hypertension, various echocardiographic indices (LVID, RAD, IVS, LVPW, AV, AMYLI score, LVEF), and echocardiographic features (asymmetric hypertrophy, granular sparkling, pericardial effusion, AR, MR, and TR severity). These predictors were subsequently evaluated using multivariate logistic regression. The analysis unveiled that the absence of hypertension, LVID, LVEF, AMYLI score, asymmetric hypertrophy, granular sparkling, pericardial effusion, AR, MR, and TR severity independently predicted CA in the LVH cohort (Table 2).

**Table 2:** Univariate and multivariate logistic regression results from the training set

	Univariate Logistic Regression		Multivariate Logistic Regression	
	OR (95%CI)	*P* Value	OR (95%CI)	*P* Value
Hypertension History	0.03 (0.02–0.05)	< 0.001	0.04 (0.021–0.073)	< 0.001
LVID	0.88 (0.86–0.91)	< 0.001	0.927 (0.878–0.977)	0.005
RVD	0.95 (0.9–1.01)	0.0865	…	…
LAD	1.02 (1-1.05)	0.0701	…	…
RAD	1.09 (1.07–1.12)	< 0.001	1.018 (0.969–1.069)	0.480
Asymmetric hypertrophy	3.46 (2.36–5.09)	< 0.001	3.729 (1.884–7.441)	< 0.001
AV	0.06 (0.03–0.12)	< 0.001	0.486 (0.154–1.507)	0.213
AMYLI Score	1.2 (1.16–1.24)	< 0.001	1.088 (1.024–1.161)	0.010
EDV index	0.98 (0.97–0.98)	< 0.001	…	…
ESV index	0.98 (0.97–0.99)	< 0.001	…	…
SV index	0.95 (0.94–0.96)	< 0.001	…	…
LVEF	0.92 (0.9–0.95)	< 0.001	0.95 (0.908–0.993)	0.024
Granular sparkling	28.79 (16.5-50.22)	< 0.001	3.111 (1.355–7.431)	0.009
Pericardial effusion (None)	1	NA	1	NA
Pericardial effusion (Small)	4.87 (3.52–6.74)	< 0.001	2.77 (1.563–4.937)	< 0.001
Pericardial effusion (Moderate to Large)	6 (2.31–15.6)	< 0.001	3.643 (0.56-21.279)	0.167
AR (None)	1	NA	1	NA
AR (Mild)	2.73 (1.95–3.81)	< 0.001	2.353 (1.278–4.361)	0.006
AR (Moderate to Severe)	0.76 (0.09–6.22)	0.7969	4.304 (0.101–82.784)	0.396
MR (None)	1	NA	1	NA
MR (Mild)	9.82 (6.85–14.09)	< 0.001	4.331 (2.347–8.141)	< 0.001
MR (Moderate to Severe)	7.95 (3.31–19.11)	< 0.001	0.893 (0.16–4.818)	0.897
TR (None)	1	NA	1	NA
TR (Mild)	10.36 (7.17–14.98)	< 0.001	3.837 (2.026–7.358)	< 0.001
TR (Moderate to Severe)	12.04 (6.46–22.45)	< 0.001	3.069 (0.845–10.987)	0.086

*LVID* Left ventricular internal diameter, *LAD* Left atrial diameter, *RVD* Right ventricular diameter, *RAD* Right atrial diameter, *IVS* Interventricular septal thickness, *LVPW* Left ventricular posterior wall thickness, *RWT* Relative wall thickness, *AV* Aortic velocity, *EDV* End-diastolic volume, *ESV* End-systolic volume, *SV* Stroke volume, *LVEF* Left ventricular ejection fraction, *LVMI* Left ventricular mass index, *AR* Aortic regurgitation, *MR* Mitral regurgitation, *TR* Tricuspid regurgitation

### Establishment and validation of the nomogram prediction model

**Fig. 2:**
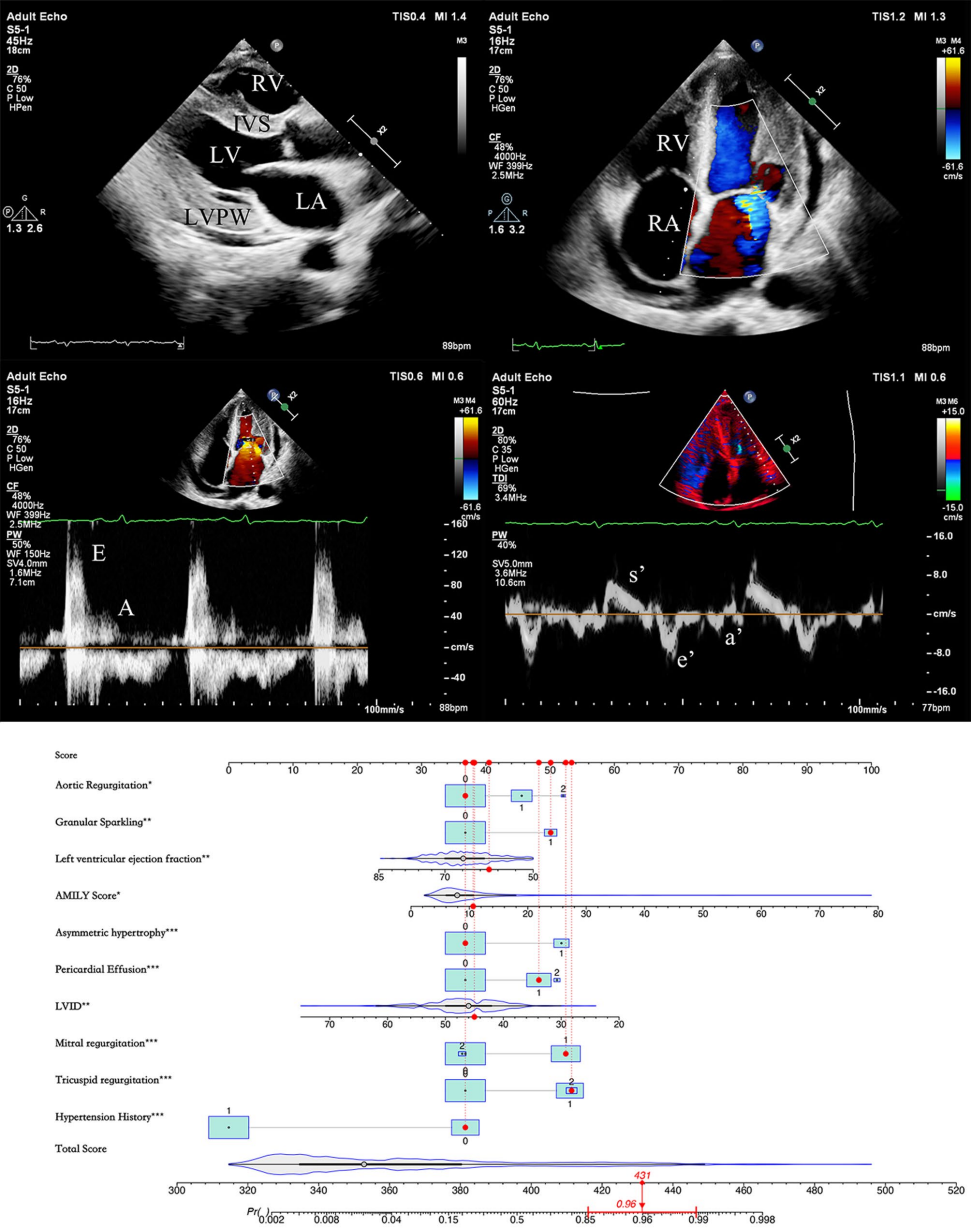
Dynamic nomogram illustration. The case depicted is of an LVH patient with no history of hypertension. Echocardiographic findings include a left ventricular end-diastolic internal diameter of 45 mm, a septal thickness of 15 mm, a posterior wall thickness of 13 mm, an E/e’ ratio of 17, an AMYLI score of 10, the presence of myocardial echo enhancement, a small pericardial effusion, absence of aortic valve regurgitation, mild mitral valve regurgitation, moderate tricuspid valve regurgitation, and a left ventricular ejection fraction of 60%. The total score calculated is 431, predicting a 96.0% probability of CA. Further diagnostic tests were recommended. The patient subsequently underwent additional examinations and was diagnosed with AL-CA

**Fig. 3:**
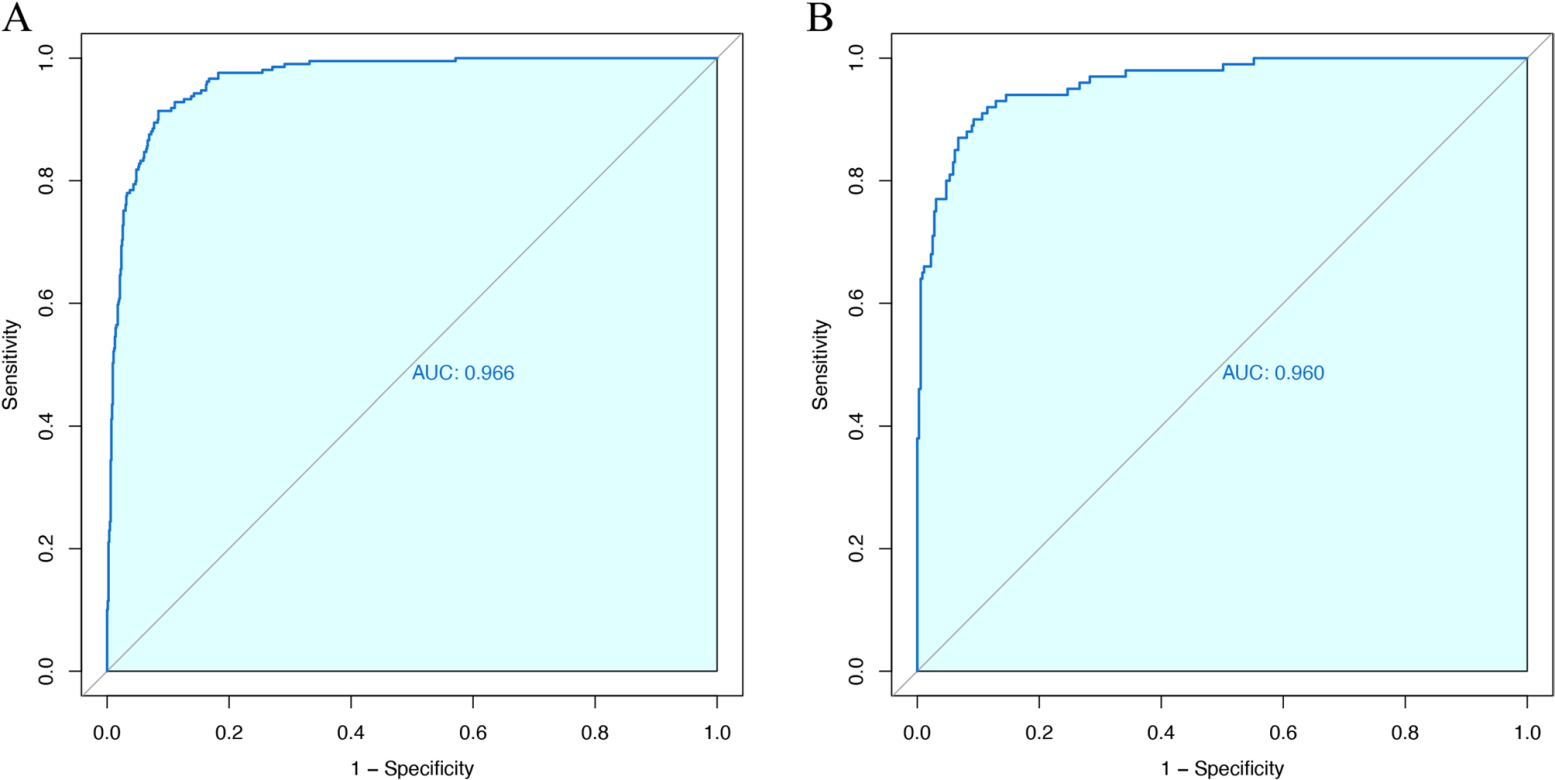
Receiver Operating Characteristic (ROC) curves of the nomogram prediction model. **A** Training set; **B **Validation set

The nomogram prediction model was developed and validated using multivariate logistic regression analysis, incorporating nine independent echocariographic predictors and one clinical predictors of CA in LVH patients. Figure 2 illustrates a representative instance of a dynamic nomogram. Model performance was evaluated using ROC curves, which demonstrated an AUC of 0.9662 for the training set and 0.9604 for the validation set (Fig. 3). At the same time, the calibration C-index was 0.9606, signifying high accuracy. The performance metrics for the training set included accuracy, specificity, sensitivity, positive predictive value (PPV), negative predictive value (NPV), and Youden index of 0.92, 0.92, 0.91, 0.73, 0.98, and 0.83, respectively. In the validation set, these metrics were 0.91, 0.91, 0.90, 0.73, 0.93, and 0.81, respectively. As anticipated, the model demonstrated superior accuracy, specificity, sensitivity, PPV, NPV, and Youden index compared to indicators used for constructing nomograms (Supplemental Table 2). While the prevalence of CA in the total cohort was 3.8%, the constructed model exhibited a PPV of 0.24 and a NPV of 0.99.

The calibration curves depicted a strong agreement between the model’s predicted probabilities and actual outcomes (Supplemental Fig. 2), with Hosmer-Lemeshow test P-values of 0.6844 and 0.3925 for the training and validation sets, respectively. DCA further validated the utility of the nomogram prediction model, which outperformed the AMYLI score across a threshold probability range of 5-80% for both sets (Supplemental Fig. 3).

**Fig. 4:**
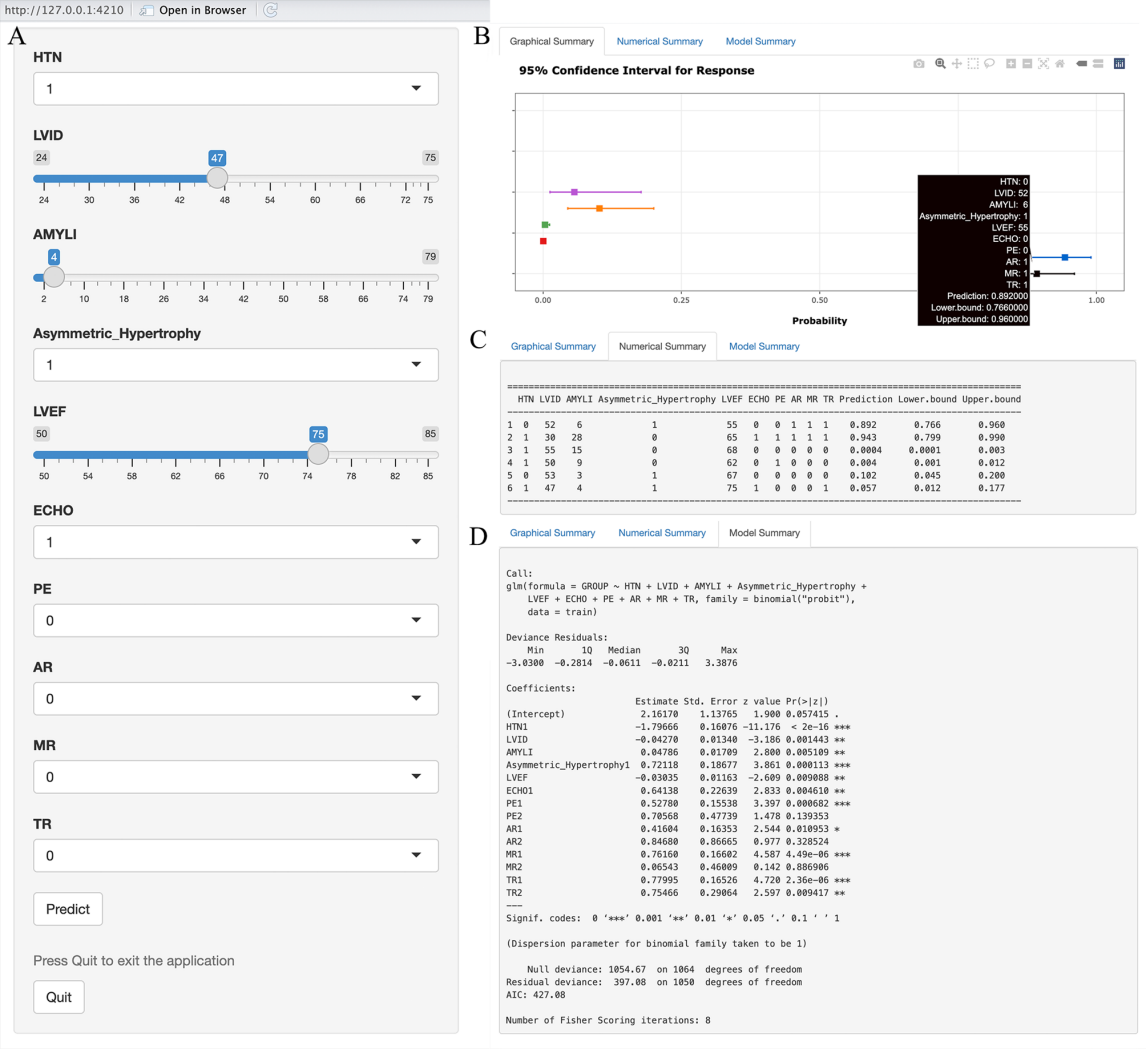
Web-based calculator interface. **A** Input interface: allows entry of patient-related variables; **B** Web calculator summary image: Predicted probability and 95% confidence interval for CA in LVH patients; **C** Data summary: Patient-related variables and predicted probability; **D** Model summary: Characteristics of variables in the model. Patients 1 and 2 were diagnosed with cardiac amyloidosis, patient 3 with uremic cardiomyopathy, patient 4 with hypertensive heart disease, and patients 5 and 6 with hypertrophic cardiomyopathy

To streamline the calculation of predicted probabilities, a web-based calculator was developed, wherein categorical, ordinal, and continuous variables can be inputted to determine the predicted probability of CA and its confidence interval, thereby informing decisions regarding further diagnostic evaluations. The interface of the web-based calculator is presented in Fig. 4, with detailed applications shown in Supplemental Figs. 4–5. The calculator can be accessed at the following URL: https://capossibility.shinyapps.io/DynNomapp/.

## Discussion

Herein, a cohort of LVH patients was established to conduct a comparative analysis of clinical and echocardiographic characteristics between patients with and without CA. Our results uncovered that the absence of a hypertension history, LVID, AMYLI score, LVEF, asymmetric hypertrophy, presence of granular sparkling, small pericardial effusion, and mild degrees of AR, MR, and TR were independent indicators of CA within the LVH demographic. Of note, the nomogram exhibited satisfactory discrimination and consistency and could accurately identify CA patients from echocardiographic data. Consequently, this model may assist in the screening of CA patients.

LVH is the most common echocardiographic feature of CA according to the ESC Guidelines [9, 11]. CA-LVH patients exhibited reduced LVID and increased RWT, indicating a hypertrophic pattern characterized by increased IVS and LVPW thicknesses. In the current study, patients with severe AS were excluded. The remaining cohort was rigorously matched for age, sex, and BSA, and absence of hypertension remained an independent predictor of CA among individuals with LVH. This finding is in line with the echocardiographic “red flags” for CA [6, 18]. It has been reported that LVH is observed in 19–48% of patients with untreated hypertension [26]. In contrast, patients with CA are more likely to exhibit hypotension [27]. This discrepancy suggests that the absence of a history of hypertension holds significant predictive value. It should be noted that the prevalence rate of hypertension history might be overestimated, as individuals with hypertension may undergo multiple echocardiographic examinations. Despite this potential overestimation, the prevalence rate of hypertension history in the study group remains significantly lower than that in the control group.

It is reported that approximately 79% of ATTR-CA cases exhibit asymmetric hypertrophy, whereas 14% of AL-CA cases show asymmetric hypertrophy [28]. The majority of patients included in our study had AL-CA, with 27.2% presenting with asymmetric hypertrophy. This proportion may be biased, potentially due to the fact that some patients in our study underwent multiple echocardiographic examinations, and our analysis was based on the number of echocardiographic examinations performed on CA patients.

We also found that amyloid deposits affect the IVS earlier, with potential earlier and more rapid thickening than the LVPW. Some patients already had markedly thickened IVS, while the LVPW was initially 8–9 mm and significantly thickened only after 3–4 years (Supplemental Fig. 6). About 30% of patients had an LVPW ≤ 11 mm at CA diagnosis, highlighting the need for further observation with a larger sample size. However, it is also worthwhile emphasizing that not all CA patients exhibit LVH [29–31], thus warranting the integration of additional echocardiographic parameters for a comprehensive diagnostic evaluation.

Combining multiple-parameter indicators in echocardiography for model construction is beneficial for the diagnosis of CA [18]. The AL and IWT scores encompass parameters such as RWT, E/e’ ratio, tricuspid annular plane systolic excursion, and left ventricular systolic longitudinal strain [7] and offer significant value in screening for CA and can effectively guide subsequent diagnostic methods and secondary imaging modalities [32–34]. Nevertheless, it should be acknowledged that right cardiac involvement typically occurs at a more advanced stage, and strain is not routinely measured in the clinical setting, thus complicating CA screening in routine clinical work. In 2020, Aimo et al. [20] exclusively selected the parameters RWT and E/e’ ratio common to both the AL score and IWT score and defined the AMYLI score as the product of these two parameters. They described that the area under the curve of the AMYLI score was comparable to that of the AL score and IWT score [20]. An AMYLI score of less than 2.36 and less than 2.22 can be used to rule out CA in individuals with suspected AL-CA and those with unexplained LVH, respectively, without the need for follow-up examinations. However, to refine the cutoff value, Aimo et al. [20] selected a lower AMYLI score, which excluded CA in only a small fraction of patients, with a positive predictive value of merely 66% for unexplained LVH. Herein, the PPV of the AMYLI score was 28%, signifying its limited utility for CA screening. By integrating additional echocardiographic parameters and features, the developed nomogram model enhanced the PPV and NPV of echocardiographic parameters for CA, signaling that a combination of these non-specific echocardiographic features might be highly indicative of CA.

Amyloid deposition on cardiac valves can induce valve thickening, potentially culminating in stenosis or regurgitation. Noteworthily, predominant valve involvement patterns in CA patients encompass AS, MR, and TR [35]. It should be noted that both AS and CA can jointly contribute to LVH [36, 37]. While patients with severe AS were excluded from this study, CA patients had lower AV compared to non-CA patients, likely ascribed to impaired cardiac function and diminished LVEF in CA patients. Aimo et al. [38] established a valve score through the evaluation of the leaflets, annuli, chordae tendineae, papillary muscles and valve functions of the aortic, mitral and tricuspid valves, and concluded that the valve score, which might be incorporated into a more comprehensive echocardiographic assessment, could at least differentiate between the two forms of CA. In the valve score, moderate and severe MR and TR are assigned a score of 1, while mild or no MR and TR are scored 0 [38]. However, in our study, moderate-to-severe MR and TR were only present in 3.6% and 12.6% of CA patients, respectively, with more than half of CA patients displaying only mild MR and TR. Compared to non-CA-LVH patients, the prevalence of mild MR and TR was higher in CA-LVH patients. Besides, while earlier research suggests that age, gender, electrocardiographic features, troponin levels, and history of carpal tunnel syndrome can aid in the diagnosis of CA [39–43], our study focused on routine echocardiographic parameters to identify indicators for CA.

Nevertheless, some limitations of this study merit acknowledgment. (1) Concerning patient enrollment, though AL-CA and ATTR-CA exhibit similar echocardiographic manifestations [6], our cohort predominantly comprised AL-CA patients, as ATTR-CA is under-diagnosed in China [44]. This diagnostic gap has resulted in a relatively low number of ATTR-CA cases with preserved LVEF in our center, as previously reported [10, 45]. Thus, the results might not be generalizable to patients with ATTR-CA. (2) Approximately 45% of the patients either received negative biopsy results, presented critical conditions such as severe infections stemming from multiple myeloma (MM) chemotherapy, experienced intolerance to the biopsy procedure, or refused the biopsy. Consequently, CA was clinically diagnosed through multidisciplinary consultations and non-invasive assessments such as CMR. Given that most patients had AL-CA and MM, and the 2023 ESC Guidelines on the Management of Cardiomyopathies [11] include a history of MM or monoclonal gammopathy of undetermined significance as screening criteria for CA, we believe the clinical diagnosis of CA for these patients is justified. (3) For model construction, 309 sets of echocardiographic datasets from 185 CA patients were utilized to increase the sample size. Although this raises the concern about potential overlap between the training and validation sets due to the repeated inclusion of data from the same patients, the echocardiographic data for each patient were collected at different time periods. As is typical in cardiovascular imaging research, this study focused on routine echocardiographic parameters to identify indicators of CA, rather than other clinical covariates such as age and sex reported by other studies [39, 42]. Despite these limitations, the nomogram demonstrated robust performance, as evidenced by the bootstrap-corrected C-index and calibration curve. Nonetheless, further external validation of other races is warranted to validate our findings and improve the applicability of the proposed model.

## Conclusion

CA is an infiltrative cardiomyopathy with a relatively low incidence. Recent advances in non-invasive diagnostic tools have markedly improved early detection. This study examined clinical history (e.g. absence of hypertension), echocardiographic parameters (LVID, LVEF, and AMYLI scores), as well as echocardiographic characteristics (asymmetric hypertrophy, granular sparkling, and the severity of pericardial effusion, AR, MR, and TR). After multivariable adjustment, these factors were confirmed as strong, independent predictors of CA in the LVH cohort. Finally, the developed nomogram demonstrated high predictive accuracy, offering the potential to enhance CA screening via echocardiography and strategically guiding subsequent evaluations.

## Supplementary Information

Supplementary Material 1.

## Data Availability

The data within this article may be shared after a reasonable request to the corresponding author.

## Abbreviations

AL-CA: Immunoglobulin light-chain cardiac amyloidosis
ATTR-CA: Transthyretin cardiac amyloidosis
AR: Aortic regurgitation
CA: Cardiac amyloidosis
CMR: Cardiac magnetic resonance
FD: Fabry disease
HCM: Hypertrophic cardiomyopathy
HHD: Hypertensive heart disease
IVS: Interventricular septal thickness
LAD: Left atrial diameter
LVEF: Left ventricular ejection fraction
LVID: Left ventricular internal diameter
LVPW: Left ventricular posterior wall
LVH: Left ventricular hypertrophy
MR: Mitral regurgitation
RAD: Right atrial diameter
RWT: Relative wall thickness
RVD: Right ventricular diameter
TR: Tricuspid regurgitation
UM: Uremic cardiomyopathy
