# Supplementary material for: Development and validation of an echocardiographic nomogram for identifying cardiac amyloidosis in patients with left ventricular hypertrophy
Source: BMC Cardiovasc Disord. 2025 Oct 27;25:767. doi: 10.1186/s12872-025-04973-5 (PMC12557862; doi:10.1186/s12872-025-04973-5)
Supplement: Supplementary file 1 — Supplementary Material 1. [file 12872_2025_4973_MOESM1_ESM.docx]

**Supplemental Table 1. Comparison between Training Set and Validation Set**

|  | **After PSM (n=1522)** | **Training set (n=1065)** | **Validation set (n=457)** | ***P Value*** |
| --- | --- | --- | --- | --- |
| **Demographic Characters** | | | | |
| Male (%) | 945 (62.1) | 664 (62.3) | 281 (61.5) | 0.796 |
| Age (years) | 61.70±14.14) | 61.39±14.27 | 62.43±13.83 | 0.191 |
| BSA (m^2^) | 1.59±0.17) | 1.59±0.17 | 1.59±0.18 | 0.423 |
| Hypertension History (%) | 1029 (67.6) | 724 (68.0) | 305 (66.7) | 0.678 |
| **Echocardiographic Parameters** | | | | |
| LVID (mm) | 46.57±5.92 | 46.56±6.07 | 46.60±5.55 | 0.900 |
| LAD (mm) | 38.11±5.92 | 38.04±5.98 | 38.29±5.78 | 0.441 |
| RVD (mm) | 21.36±2.81 | 21.31±2.88 | 21.47±2.66 | 0.315 |
| RAD (mm) | 36.90±6.27 | 36.75±6.18 | 37.25±6.45 | 0.162 |
| IVS (mm) | 14.08±2.16 | 14.08±2.17 | 14.09±2.15 | 0.916 |
| LVPW（mm） | 12.35±1.60 | 12.38±1.57 | 12.29±1.68 | 0.325 |
| IVS/LVPW ratio | 1.15±0.23 | 1.15±0.23 | 1.16±0.23 | 0.421 |
| Asymmetric hypertrophy (%) | 208 (13.7) | 135 (12.7) | 73 (16.0) | 0.102 |
| RWT | 0.54±0.11 | 0.54±0.11 | 0.54±0.11 | 0.383 |
| AV (m/s) | 1.45±0.26 | 1.45±0.26 | 1.44±0.25 | 0.872 |
| E/e' | 15.18 [12.00, 20.00] | 15.00 [12.00, 20.00] | 16.00 [12.00, 20.00] | 0.113 |
| AMYLI Score | 7.98 [6.04, 10.92] | 7.92 [6.00, 10.80] | 8.20 [6.24, 11.25] | 0.259 |
| EDV index (ml/m^2^) | 64.53±18.64 | 64.59±19.03 | 64.39±17.73 | 0.849 |
| ESV index (ml/m^2^) | 22.59±8.75 | 22.61±8.88 | 22.55±8.43 | 0.908 |
| SV index (ml/m^2^) | 41.94±11.71 | 41.98±11.92 | 41.84±11.24 | 0.828 |
| LVEF (%) | 65.35±6.42 | 65.38±6.41 | 65.30±6.44 | 0.835 |
| LVMI (g/m^2^) | 148.55 [129.06, 174.23] | 148.34 [128.49, 175.52] | 149.54 [130.48, 170.02] | 0.911 |
| **Echocardiographic Characters** | | | | |
| Granular sparkling (%) | 131 (8.6) | 94 (8.8) | 37 (8.1) | 0.715 |
| Pericardial effusion (%) | ... | ... | ... | 0.586 |
| None | 1089 (71.6) | 765 (71.8) | 324 (70.9) | ... |
| Mild | 410 (26.9) | 282 (26.5) | 128 (28.0) | ... |
| Moderate to Severe | 23 (1.5) | 18 (1.7) | 5 (1.1) | ... |
| AR（%） | ... | ... | ... | 0.617 |
| None | 1180 (77.5) | 833 (78.2) | 347 (75.9) | ... |
| Mild | 330 (21.7) | 224 (21.0) | 106 (23.2) | ... |
| Moderate to Severe | 12 ( 0.8) | 8 (0.8) | 4 (0.9) | ... |
| MR（%） | ... | ... | ... | 0.478 |
| None | 979 (64.3) | 684 (64.2) | 295 (64.6) | ... |
| Mild | 513 (33.7) | 357 (33.5) | 156 (34.1) | ... |
| Moderate to Severe | 30 (2.0) | 24 (2.3) | 6 (1.3) | ... |
| TR（%） | ... | ... | ... | 0.740 |
| None | 993 (65.2) | 698 (65.5) | 295 (64.6) | ... |
| Mild | 459 (30.2) | 316 (29.7) | 143 (31.3) | ... |
| Moderate to Severe | 70 (4.6) | 51 (4.8) | 19 (4.2) | ... |

BSA: body surface area; LVID: left ventricular internal diameter; LAD: left atrial diameter; RVD: right ventricular diameter; RAD: right atrial diameter; IVS: interventricular septal thickness; LVPW: left ventricular posterior wall thickness; RWT: relative wall thickness; AV: aortic velocity; EDV: end-diastolic volume; ESV: end-systolic volume; SV: stroke volume; LVEF: left ventricular ejection fraction; LVMI: left ventricular mass index; AR: aortic regurgitation; MR: mitral regurgitation; TR: tricuspid regurgitation.

**Supplemental Table 2. Comparison of Indicators for Constructing Nomograms in Predicting CA in LVH Population.**

|  | **Acuuracy** | **Specificity** | **Sensitivity** | **Positive Predictive Value** | **Negative Predictive Value** | **Youden Index** |
| --- | --- | --- | --- | --- | --- | --- |
| Training Set | 0.92 | 0.92 | 0.91 | 0.73 | 0.98 | 0.83 |
| Validation Set | 0.91 | 0.91 | 0.90 | 0.73 | 0.93 | 0.81 |
| AMYLI Score | 0.79 | 0.88 | 0.45 | 0.48 | 0.86 | 0.33 |
| RWT | 0.81 | 0.89 | 0.46 | 0.53 | 0.87 | 0.36 |
| E/e' | 0.67 | 0.69 | 0.59 | 0.33 | 0.87 | 0.28 |
| LVID | 0.63 | 0.62 | 0.66 | 0.31 | 0.88 | 0.28 |
| LVEF | 0.61 | 0.62 | 0.59 | 0.28 | 0.86 | 0.21 |
| Granular sparkling | 0.85 | 0.85 | 0.80 | 0.34 | 0.98 | 0.65 |
| Pericardial effusion | 0.72 | 0.87 | 0.39 | 0.54 | 0.78 | 0.26 |
| AR | 0.71 | 0.84 | 0.35 | 0.39 | 0.82 | 0.19 |
| MR | 0.74 | 0.93 | 0.44 | 0.77 | 0.75 | 0.37 |
| TR | 0.75 | 0.93 | 0.46 | 0.78 | 0.76 | 0.39 |

LVID: left ventricular internal diameter; RWT: relative wall thickness; LVPW: left ventricular posterior wall thickness; AR: aortic regurgitation; MR: mitral regurgitation; TR: tricuspid regurgitation.

**
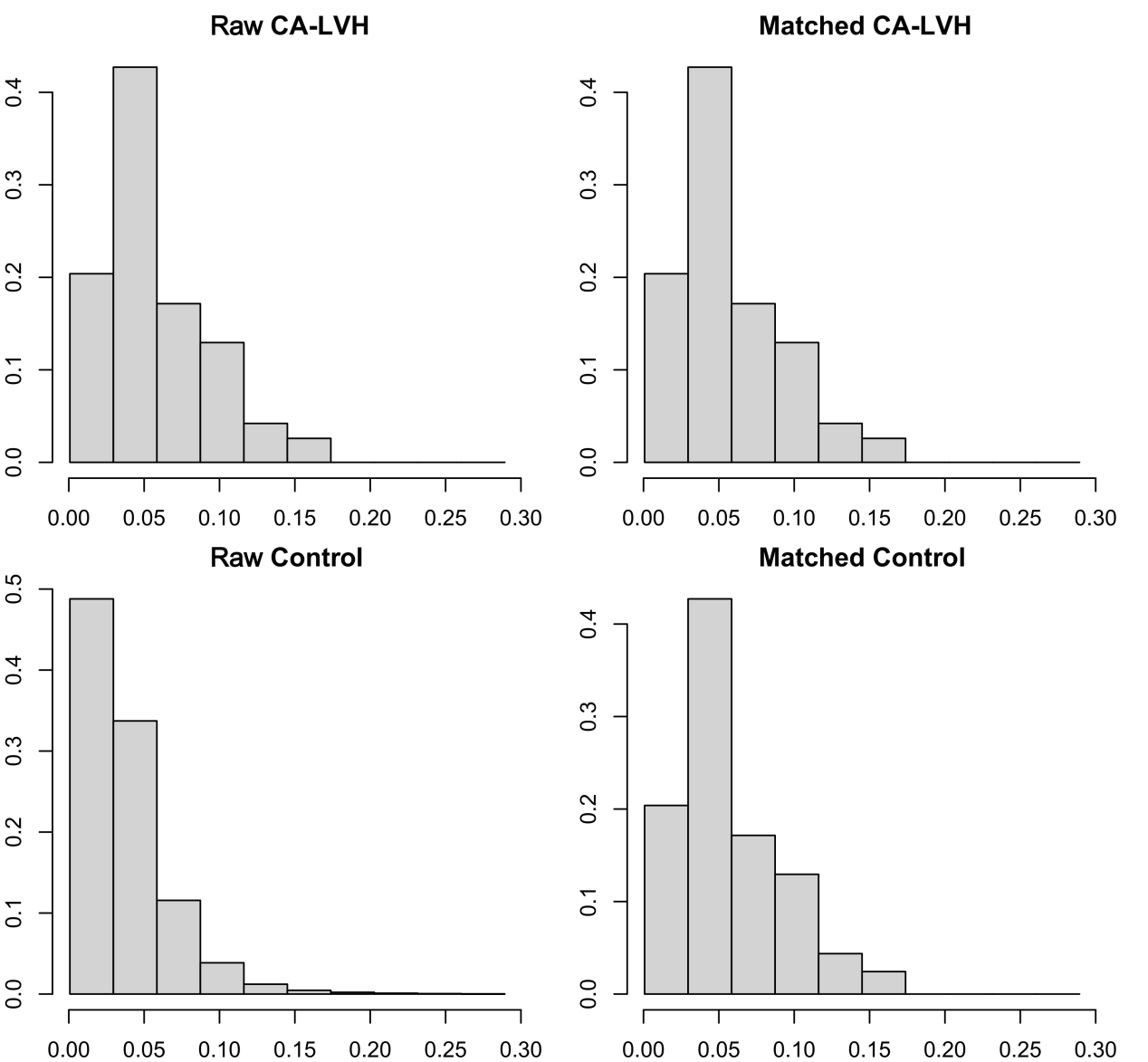
**

**Supplemental Figure 1. Results of propensity score.** The left side represents the unmatched histogram, and the right side displays the histogram after matching.

**
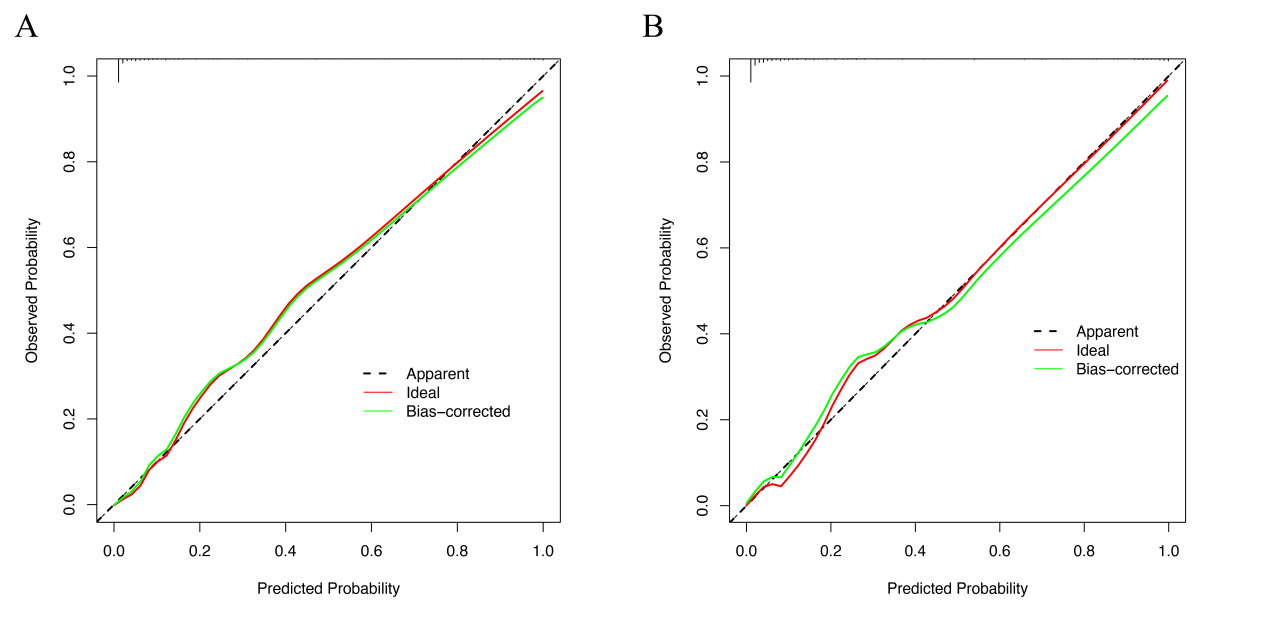
**

**Supplemental Figure 2. Calibration curve of the nomogram prediction model.** The X-axis represents the predicted probabilities obtained from the nomogram, and the Y-axis represents the actual probabilities of CA in the LVH population. The black dotted line denotes perfect agreement between predicted and actual probabilities. The red solid line represents the performance of the nomogram for the entire cohort, while the green line represents the bias-corrected prediction performance through bootstrap resampling (1,000 repetitions). A. Training set; B. Validation set.

**
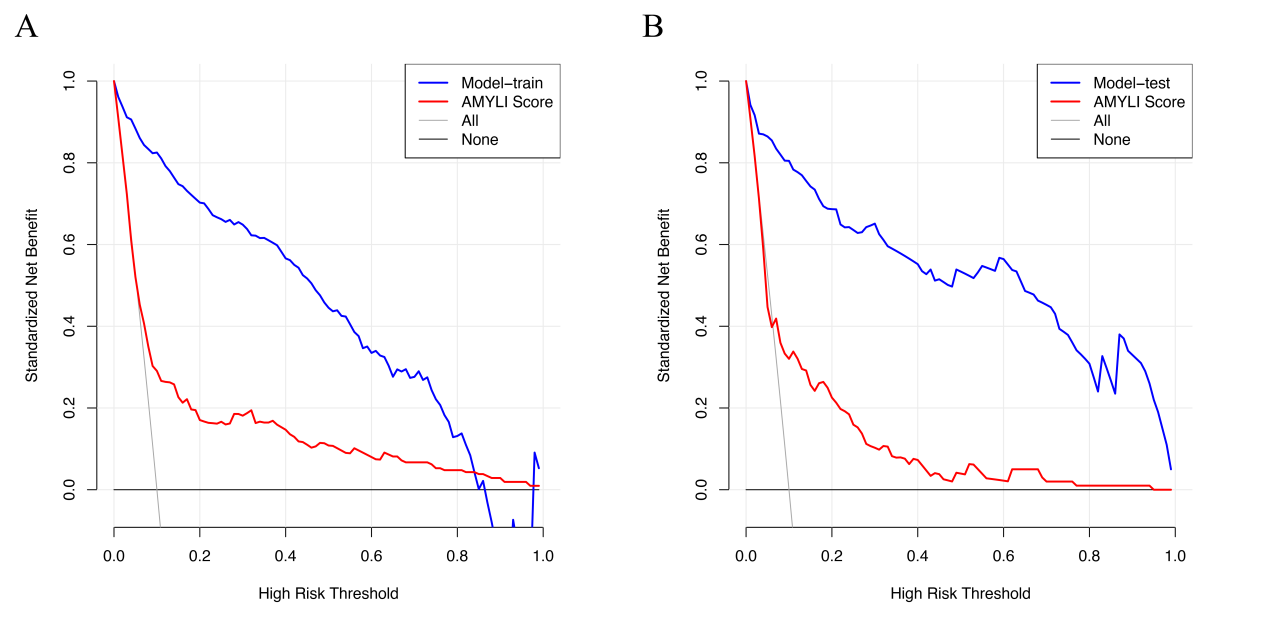
**

**Supplemental Figure 3. Decision curve of the nomogram prediction model.** The X-axis represents the potential risk threshold for CA in the LVH population, and the Y-axis represents the net benefit of the nomogram. The gray sloping line is the clinical net-benefit curve of the diagnostic model if all patients in the LVH population are diagnosed with CA. The black horizontal line represents the clinical net-benefit curve of the diagnostic model when no patients in the LVH population are diagnosed with CA (this line corresponds to the 0 point on the vertical axis, indicating that the clinical net-benefit of the diagnostic model is 0 when none of the patients in the LVH population are diagnosed with CA). The blue curve illustrates the decision curve of the nomogram prediction model, while the red curve delineates the decision curve of the AMYLY score. A. Training set; B. Validation set.


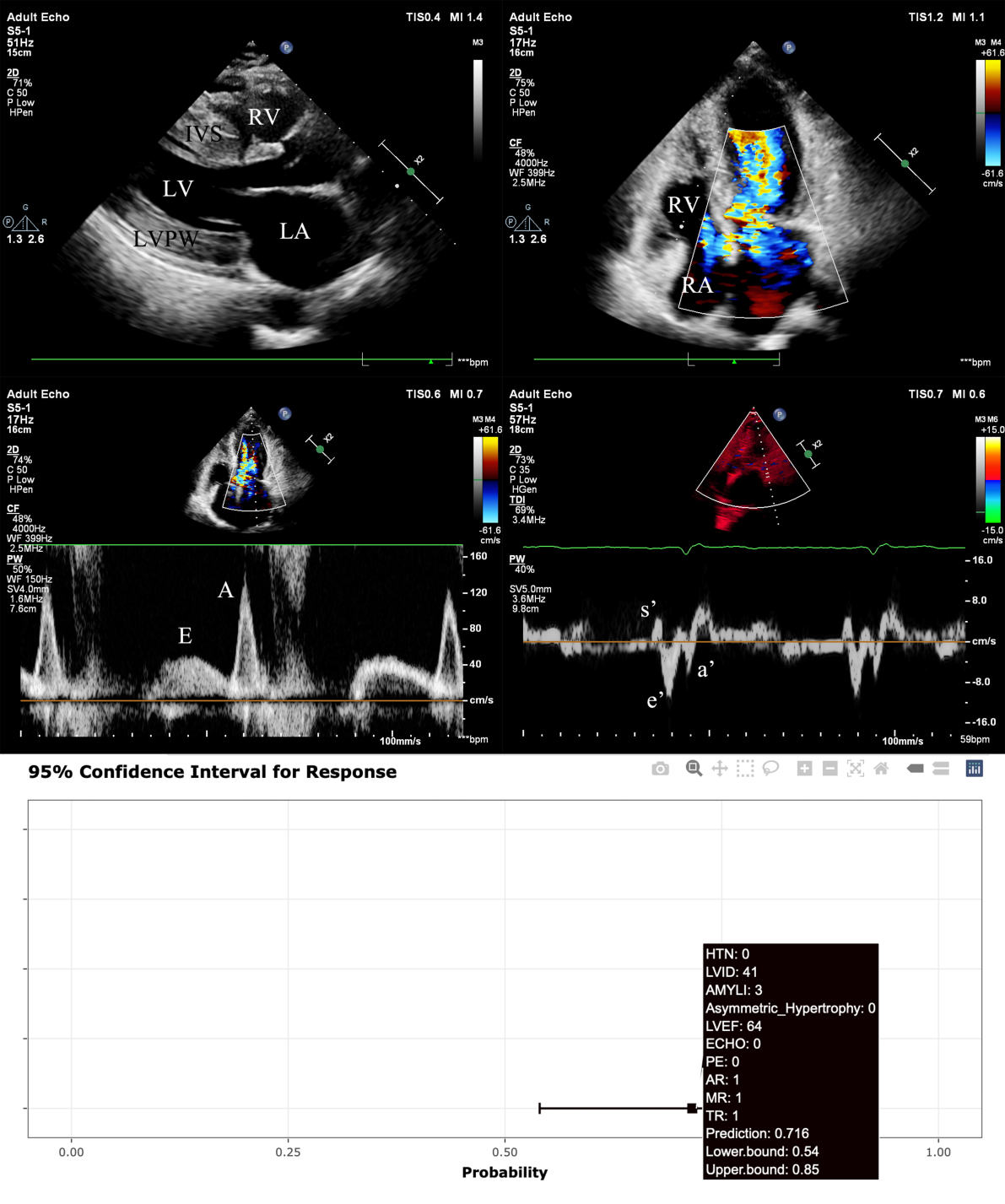


**Supplemental Figure 4. Web-based calculator illustration 1.** For an LVH patient without a history of hypertension, echocardiography findings indicated that the left ventricular end-diastolic diameter was 41 mm, the interventricular septum thickness was 16 mm, the left ventricular posterior wall thickness was 14 mm, the E/e' ratio was 4.5, and the AMYLI score was 3 points. No evidence of myocardial echo enhancement or pericardial effusion was noted. Mild aortic regurgitation, mild-to-moderate mitral regurgitation, and mild tricuspid regurgitation were observed. The left ventricular ejection fraction was 64%. The predicted probability of this patient having CA was 71.6%, warranting further examinations for confirmation. Eventually, this patient was diagnosed with HCM after completing subsequent examinations.


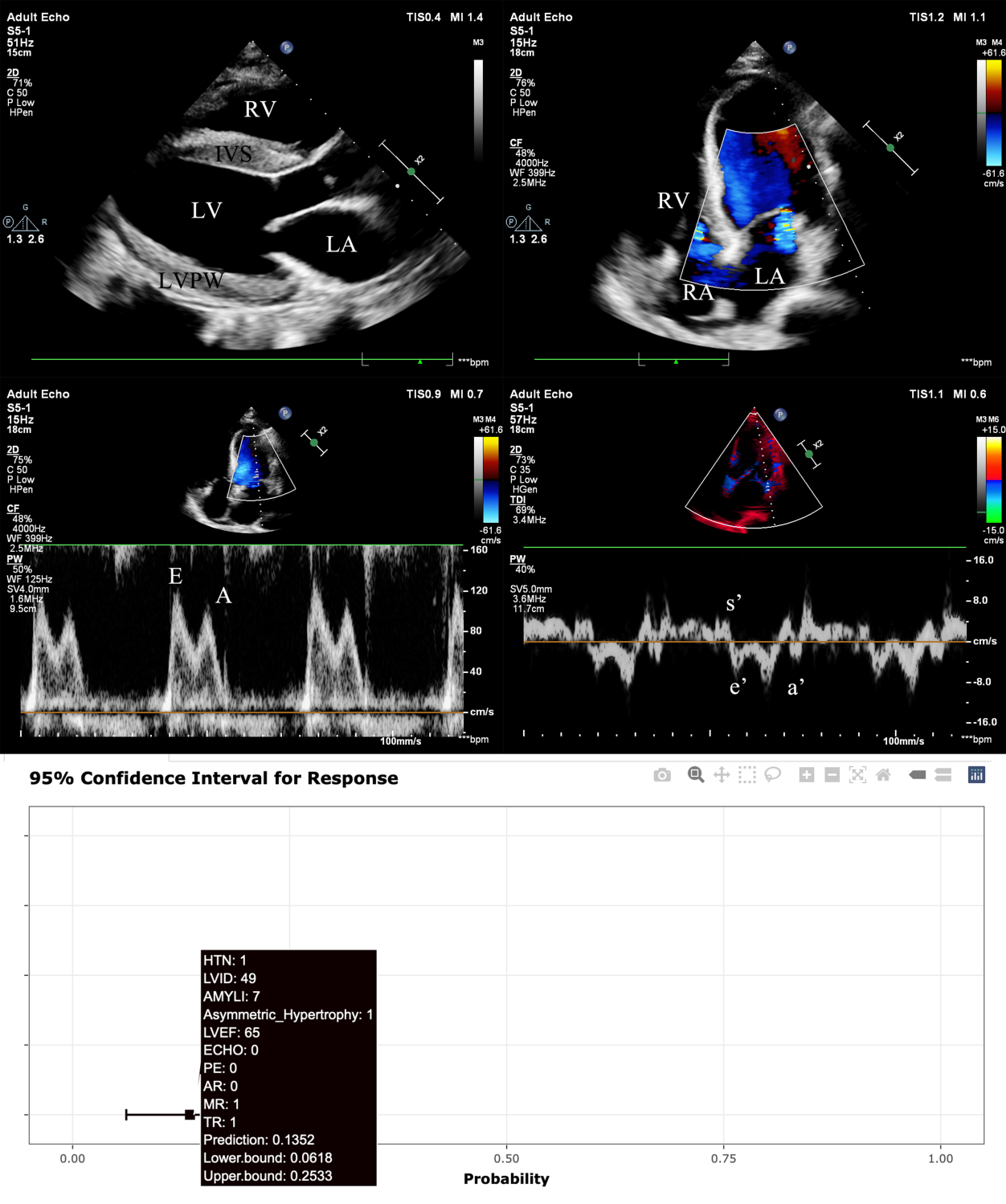


**Supplemental Figure 5. Web-based calculator illustration 2.** For an LVH patient with a history of renal hypertension, echocardiography findings revealed a left ventricular end-diastolic diameter of 49 mm, an interventricular septum thickness of 18 mm, a left ventricular posterior wall thickness of 12 mm, an E/e' ratio of 15, and an AMYLI score of 7. No evidence of myocardial echo enhancement, pericardial effusion, or aortic regurgitation was observed. Mild mitral and tricuspid regurgitation were noted, and the left ventricular ejection fraction was 65%. The predicted probability of this patient having CA was 13.5%. Eventually, this patient was diagnosed with UM after completing subsequent examinations.

**
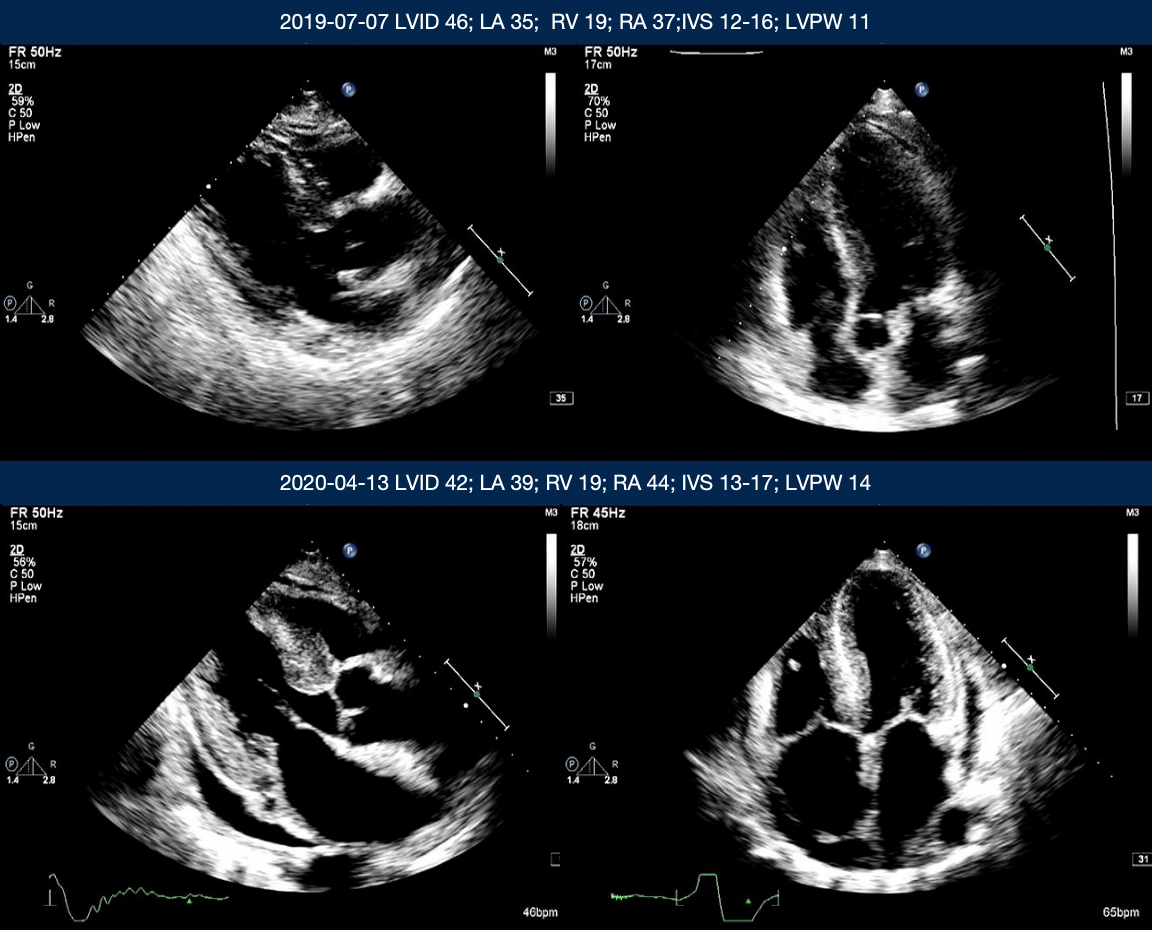
**

**Supplemental Figure 6. Illustration of echocardiograms from a patient with cardiac amyloidosis (CA) at different time points.**
